# Comprehensive Assessment of Host Responses to Ionizing Radiation by Nuclear Factor-κB Bioluminescence Imaging-Guided Transcriptomic Analysis

**DOI:** 10.1371/journal.pone.0023682

**Published:** 2011-08-24

**Authors:** Chung-Ta Chang, Ho Lin, Tin-Yun Ho, Chia-Cheng Li, Hsin-Yi Lo, Shih-Lu Wu, Yi-Fang Huang, Ji-An Liang, Chien-Yun Hsiang

**Affiliations:** 1 Emergency Medicine Department, Far Eastern Memorial Hospital, Taipei, Taiwan; 2 Department of Life Sciences, National Chung Hsing University, Taichung, Taiwan; 3 Graduate Institute of Chinese Medicine, China Medical University, Taichung, Taiwan; 4 Department of Nuclear Medicine, China Medical University Hospital, Taichung, Taiwan; 5 Department of Biochemistry, China Medical University, Taichung, Taiwan; 6 Department of Prosthodontics, Dental Section, Chang Gung Memorial Hospital, Taoyuan, Taiwan; 7 Department of Radiation Therapy and Oncology, China Medical University Hospital, Taichung, Taiwan; 8 Department of Microbiology, China Medical University, Taichung, Taiwan; Emory University, United States of America

## Abstract

The aim of this study was to analyze the host responses to ionizing radiation by nuclear factor-κB (NF-κB) bioluminescence imaging-guided transcriptomic tool. Transgenic mice carrying the NF-κB-driven luciferase gene were exposed to a single dose of 8.5 Gy total-body irradiation. *In vivo* imaging showed that a maximal NF-κB-dependent bioluminescent intensity was observed at 3 h after irradiation and *ex vivo* imaging showed that liver, intestine, and brain displayed strong NF-κB activations. Microarray analysis of these organs showed that irradiation altered gene expression signatures in an organ-specific manner and several pathways associated with metabolism and immune system were significantly altered. Additionally, the upregulation of fatty acid binding protein 4, serum amyloid A2, and serum amyloid A3 genes, which participate in both inflammation and lipid metabolism, suggested that irradiation might affect the cross pathways of metabolism and inflammation. Moreover, the alteration of chemokine (CC-motif) ligand 5, chemokine (CC-motif) ligand 20, and Jagged 1 genes, which are involved in the inflammation and enterocyte proliferation, suggested that these genes might be involved in the radiation enteropathy. In conclusion, this report describes the comprehensive evaluation of host responses to ionizing radiation. Our findings provide the fundamental information about the *in vivo* NF-κB activity and transcriptomic pattern after irradiation. Moreover, novel targets involved in radiation injury are also suggested.

## Introduction

Radiation therapy is used commonly for solid cancers. More than 50% of patients with cancers receive radiation as a component of their treatment. Although improvements in radiation therapy have led to a reduction in the volume of normal tissue irradiated, injury to central nervous system, gastrointestinal tract, and kidney occurs commonly in patients undergoing cancer therapy. It has been known that ionizing irradiating normal tissues leads to tissue damages [1], [2]. Ionizing radiation causes DNA damage by breaking DNA strands or generating reactive oxidative species. Reactive oxidative species further induce oxidative stress and subsequently elicit cellular defense mechanisms, such as cell cycle arrest, DNA repair, inflammation, and activation of transcription factors like nuclear factor-κB (NF-κB) [3]–[5].

NF-κB is an inducible transcription factor that consists of heterodimers of RelA (p65), c-Rel, RelB, p50/NF-κB1, and p52/NF-κB2. NF-κB is a central coordinator of innate and adaptive immune responses. It also plays critical roles in the development of cancer, regulation of cell apoptosis, and control of cell cycle [6]–[8]. NF-κB activity is induced by a large variety of signals, which typically include cytokines, mitogens, environmental particles, toxic metals, intracellular stresses, pathogen products, ultraviolet light, and ionizing radiation [5]. This property suggests that NF-κB may function as a sensor to detect cell responses to various stimuli.

Host-ionizing radiation interaction is a very complex process. Host responses to ionizing radiation control the performance of therapeutics. Several studies have analyzed the long-term or short-term effects of ionizing radiation on individual organs by histological examination, DNA microarray, or gel shift assay [9]–[13]. However, examining the responses of individual organs may not fulfill the global evaluation of host response to ionizing radiation. In our previous study, we have applied NF-κB bioluminescence imaging-guided transcriptomic analysis to assess the host-biomaterial interaction *in vivo* [14]. Transgenic mice carrying the NF-κB-driven luciferase gene were used to monitor the host response after implantation, and the host-biomaterial interaction was further analyzed by transcriptomic tools. In this study, we applied such a platform to evaluate the host reaction responsive to radiation exposure. Our findings provided the fundamental impacts into the radiation-affected NF-κB bioluminescent imaging and transcriptomic pattern in the whole body. Additionally, novel targets involved in radiation injury were also suggested.

## Materials and Methods

### Materials

d-Luciferin was purchased from Xenogen (Hopkinton, MA, USA) and dissolved in phosphate-buffered saline (137 mM NaCl, 1.4 mM KH_2_PO_4_, 4.3 mM Na_2_HPO_4_, 2.7 mM KCl, pH 7.2) at 15 mg/ml. Mouse monoclonal antibody against NF-κB was purchased from Chemicon (Temecula, CA, USA). Rat monoclonal antibody against F4/80, and rabbit polyclonal antibodies against phospholipase A2 (PLA2) and olig2 were purchased from abcam® (Cambridge, UK). Biotinylated rabbit anti-mouse secondary antibody was purchased from Zymed Laboratories (Carlsbad, CA, USA). Alexa Fluor® 488-conjugated goat anti-mouse IgG and Alexa Fluor® 644-conjugated goat anti-rat and anti-rabbit IgG were purchased from Invitrogen (Eugene, OR, USA).

### Animal experiments

Mouse experiments were conducted under ethics approval from China Medical University Animal Ethics Committee (Permit Number: 97-42-N). Transgenic mice carrying the NF-κB-driven luciferase gene were constructed as described previously [15].

Male transgenic mice (6 to 8 weeks old) were exposed to a single dose of whole-body X-ray at a dose rate of 4 Gy/min (Clinac® 6EX medical linear accelerator, Varian, Palo Alto, CA, USA). Mice were imaged at indicated periods after irradiation with 8.5 Gy. RNAs were extracted at 3 h after irradiation.

### *In vivo* and *ex vivo* imaging of luciferase activity

For *in vivo* imaging, mice were anesthetized with isoflurane and injected intraperitoneally with 150 mg luciferin/kg body weight. Five minutes later, mice were placed in the chamber and imaged for 1 min with the camera set at the highest sensitivity by IVIS Imaging System® 200 Series (Xenogen). Photons emitted from the body were quantified using Living Image® software (Xenogen). Signal intensity was quantified as the sum of all detected photon counts from the whole body and presented as photons/sec. For *ex vivo* imaging, mice were anesthetized and injected with luciferin intraperitoneally. Five minutes later, mice were sacrificed and tissues were rapidly removed. Tissues were placed in the IVIS system and imaged with the same setting used for *in vivo* studies. Signal intensity was quantified as the sum of all detected photon counts per second within the region of interest after subtracting the background luminescence and presented as photons/sec/cm^2^/steradian (photons/sec/cm^2^/sr).

### Histological analysis

Organs were removed, fixed in 10% (v/v) phosphate-buffered formalin solution for 2 d, rinsed in saline, and dehydrated in a series of graded alcohols (50% (v/v), 70% (v/v), and 95% (v/v)) for 30 min each. Samples were then embedded in paraffin, cut into 5-µm sections, and stained with hematoxylin and eosin (H&E). The stained sections of each sample were examined using light microscopy.

### Immunohistochemical staining and immunofluorescence staining

Immunohistochemical staining was performed as described previously [15]. Sections of 5 µm were deparaffinized in xylene and rehydrated in graded alcohol. Antigen retrieval was performed with sodium citrate buffer (10 mM sodium citrate, 0.05% Tween 20, pH 6.0) at 60°C overnight. The nonspecific binding was blocked with 1% (w/v) bovine serum albumin at room temperature for 1 h. Sections were incubated with antibodies against NF-κB at 1∶50 dilution, F4/80 at 1∶100 dilution, PLA2 at 1∶100 dilution, and olig2 at 1∶1000 dilution at 4°C overnight. Sections were then incubated with Alexa Fluor® 488- or Alexa Fluor® 644-conjugated secondary antibodies at room temperature for 1 h. Finally, the immunostained cells were visualized under a confocal laser scanning microscope (model TCS SP2, Leica, Wetzlar, Germany).

### Total RNA isolation

Total RNAs were extracted from individual organs using RNeasy Mini kit (Qiagen, Valencia, CA, USA) and quantified using the spectrophotometer (Beckman Coulter, Fullerton, CA, USA). Samples with A260/A280 ratios greater than 1.8 were further evaluated using Agilent 2100 bioanalyzer (Agilent Technologies, Santa Clara, CA, USA). The RNA sample with a RNA integrity number greater than 7.0 was accepted for microarray analysis.

### Microarray analysis

Microarray analysis was performed as described previously [14], [16]. Briefly, fluorescence-labeled RNA targets were prepared from 5 µg of total RNA using MessageAmp™ aRNA kit (Ambion, Austin, TX, USA) and cyanine (Cy5) dye (Amersham Pharmacia, Piscataway, NJ, USA). Fluorescent targets were hybridized to the Mouse Whole Genome OneArray™ (Phalanx Biotech Group, Hsinchu, Taiwan) and scanned by an Axon 4000 scanner (Molecular Devices, Sunnyvale, CA, USA). Six replicates from six independent mice were performed. The Cy5 fluorescent intensity of each spot was analyzed by genepix 4.1 software (Molecular Devices). The signal intensity of each spot was corrected by subtracting background signals in the surrounding. We filtered out spots that signal-to-noise ratio was less than 1 or control probes. Spots that passed these criteria were normalized by R program [17]. The fold changes of genes were calculated by dividing the normalized signal intensities of genes in irradiation-treated mice by those in untreated mice. Genes with fold changes >1.8 or <−1.8 were analyzed by Kyoto Encyclopedia of Genes and Genomes (KEGG) pathways on the Gene Ontology Tree Machine web site (http://bioinfo.vanderbilt.edu/gotm/), a web-based and tree-based data mining environment for gene sets [18]. We used the geneSetTest function implemented in the limma package to test significant KEGG pathways. Furthermore, genes with fold changes >1.8 or <−1.8 were analyzed by gene ontology (GO) on the Gene Ontology Tree Machine web site. We used the WebGestalt tool to test significant GO terms [19]. Microarray data is MIAME compliant and the raw data has been deposited in a MIAME compliant database, the accession number is GSE25208.

### Quantitative real-time PCR (qPCR)

The expression levels of chemokine (CC-motif) ligand 5 (Ccl5), Ccl20, Jagged1, serum amyloid A2 (SAA2), and SAA3 genes were validated by qPCR. RNA samples were reverse-transcribed for 2 h at 37°C with High Capacity cDNA Reverse Transcription Kit (Applied Biosystems, Foster City, CA, USA). qPCR was performed by using 1 µl of cDNA, 2× SYBR Green PCR Master Mix (Applied Biosystems), and 200 nM of forward and reverse primers. The reaction condition was followed: 10 min at 95°C, and 40 cycles of 15 sec at 95°C, 1 min at 60°C. Each assay was run on an Applied Biosystems 7300 Real-Time PCR system in triplicates. Fold changes were calculated using the comparative C_T_ method. The primer set for each gene is followed: Ccl5 forward, 5′-ATATGGCTCGGACACCACTC-3′; Ccl5 reverse, 5′-AACACGACTGCAAGATTGGAG-3′; Ccl20 forward, 5′-ATACAGACGCCTCTTCCTTCC-3′; Ccl20 reverse, 5′-CAGCCCTTTTCACCCAGTTC-3′; Jagged1 forward, 5′-TAGTAAACGGGATGGAAACAGC-3′; Jagged1 reverse, 5′-CAGCAGAGGAACCAGGAAATC-3′; SAA2 forward, 5′- AATCAGTGATGCAAGAGAGAGC-3′; SAA2 reverse, 5′- CAGTATTTGGCAGGCAGTCC-3′; SAA3 forward, 5′- CCTGGGCTGCTAAAGTCATC-3′; SAA3 reverse, 5′- CACTCATTGGCAAACTGGTCAG-3′; glyceraldehyde-3-phosphate dehydrogenase (GAPDH) forward, 5′-ACACCCACTCCTCCACCTTT-3′ ; GAPDH reverse, 5′-TAGCCAAATTCGTTGTCATACC-3′.

### Statistic analysis

Data were presented as mean ± standard error. For imaging data, Student's *t*-test was used for comparisons between two experiments. A value of *p*<0.05 was considered statistically significant.

## Results

### Assessment of the NF-κB-driven bioluminescent signal in ionizing radiation-exposed mice by *in vivo* and *ex vivo* imaging

Transgenic mice constructed here contained the luciferase gene driven by a promoter with five NF-κB-responsive elements. Therefore, the luciferase activity reflected the NF-κB *trans*-activity [14], [15].

**Figure 1:**
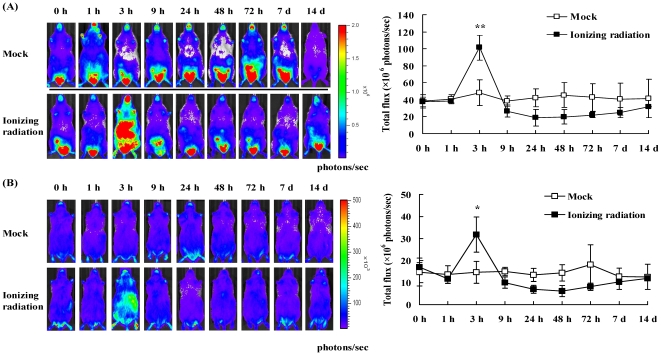
NF-κB-dependent bioluminescence in living mice. Transgenic mice were exposed to ionizing radiation and imaged in ventral (A) and dorsal positions (B) at indicated periods. *In vivo* imaging was shown on the left panel. The color overlay on the image represents the photons/sec emitted from the animal, as indicated by the color scale. Photos are representative images (*n* = 10). Quantification of photon emission from the whole animal was shown on the right panel. Shown is the total photon flux plotted over time. Values are mean ± standard error (*n* = 10). * *p*<0.05, ** *p*<0.01, compared with mock.

Male transgenic mice were exposed to ionizing radiation and then imaged at different periods. As shown in Figure 1 and Figure S1, NF-κB-driven bioluminescent signals from ventral and dorsal areas of untreated mice were unchanged over time, while luminescence from irradiation-exposed mice reached a maximal intensity at 3 h. At 3 h, a diffuse luminescence was detected throughout the body and a strong signal was emitted in the abdominal region. These findings indicated that ionizing radiation induced an acute activation of NF-κB at 3 h.

**Figure 2:**
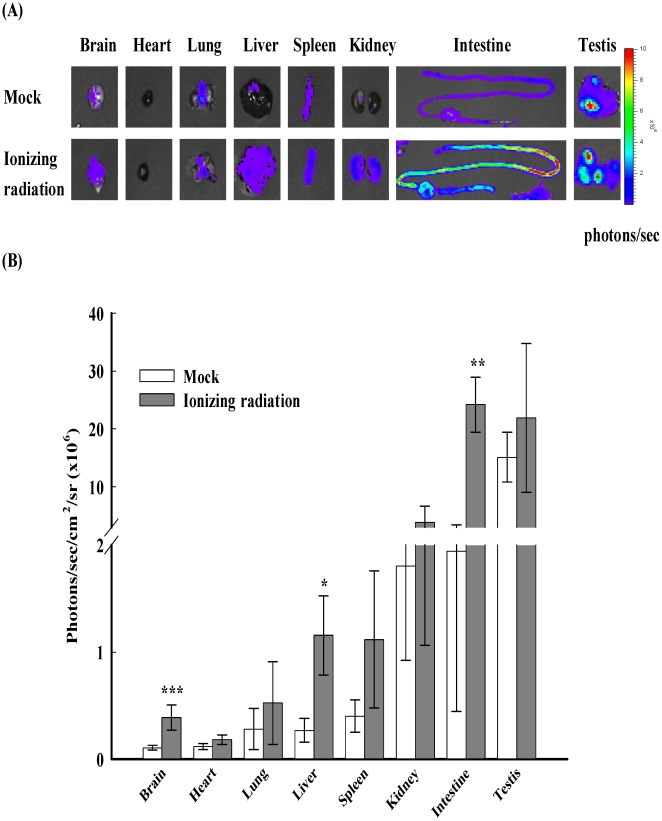
NF-κB-dependent bioluminescence in individual organs. Transgenic mice were exposed to ionizing radiation. Three hours later, mice were sacrificed and organs were subjected to image. (A) *Ex vivo* imaging. The color overlay on the image represents the photons/sec emitted from the organ, as indicated by the color scale. Photos are representative images (*n* = 10). (B) Quantification of photon emission from the organ. Values are mean ± standard error (*n* = 10). * *p*<0.05, ** *p*<0.01, *** *p*<0.001, compared with mock.

Next, we would like to analyze the bioluminescent signals of individual organs after irradiation. Transgenic mice were exposed to 8.5 Gy and sacrificed at 3 h. In comparison with mock, luminescence from most organs was increased after irradiation (Figure 2). These findings suggested that NF-κB activities in most organs were affected by irradiation. Irradiation significantly increased NF-κB-dependent luminescent signals in brain, liver, and intestine, with a 3.7-, 4.3-, and 13.5-fold induction, respectively. Irradiation moderately increased the luminescent signals in heart, lung, spleen, kidney, and testis, with a 1.6-, 1.9-, 2.8-, 2.1-, and 1.4-fold induction, respectively. These data indicated that ionizing radiation induced a strong activation of NF-κB-driven luminescence in brain, liver, and intestine.

### Immunohistochemical staining and immunofluorescence staining of organs after irradiation

**Figure 3:**
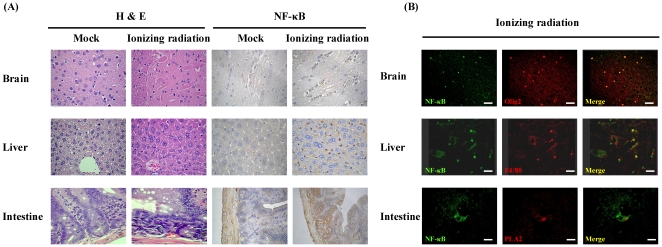
Histological examination, immunohistochemical staining, and immunofluorescence staining of organs exposed to ionizing radiation. (A) Histological examination and immunohistochemical staining. Transgenic mice were exposed to irradiation. Three hours later, mice were sacrificed, organs were excised, and the sections were stained with H&E or by immunohistochemistry using antibody against NF-κB (400× magnification). Photos are representative images (*n* = 6). (B) Immunofluorescence staining. Sections from irradiation-exposed organs were stained with antibodies against NF-κB (green) , olig2 (ref), F4/80 (ref), or PLA2 (ref). Overlap of markers appears as yellow color in the right panels. Scale bars = 10 µm. Photos are representative images (*n* = 3).

It has been known that irradiating normal tissues leads to tissue damage, such as inflammation, fibrosis, or necrosis [2], [3]. Histological examination showed that inflammation, characterized by the infiltration of immune cells, hemorrhage, and the accumulation of fluid, was evoked by irradiation at 3 h in brain, liver, and intestine (Figure 3(A)). Immunohistochemical staining with antibody against NF-κB p65 subunit revealed that, in comparison with mock, there were many brown p65-reactive cells in irradiation-exposed organs. In the liver, p65-negative hepatocytes were surrounded by many brown p65-reactive Kupffer cells and endothelial cells. Additionally, brown-p65-reactive cells in the intestine gland and brown-p65-reactive oligodendrocytes in the brain were observed. To further characterize the NF-κB-responsive cell types in each organ, we performed double immunofluorescence staining. Sections were stained for F4/80, PLA2, and olig2 to identify Kupffer cells in the liver, Paneth cells in the intestine, and oligodendrocytes in the brain, respectively [20]–[22]. Figure 3(B) shows that NF-κB (green fluorescence) was colocalized with markers (ref fluorescence) for Kupffer cells, Paneth cells, or oligodendrocytes. These results indicated that ionizing radiation evoked acute inflammatory response in brain, liver, and intestine at 3 h. Moreover, irradiation induced NF-κB activation of specific cell types in these organs.

### Transcriptomic analysis of organs after irradiation

By *ex vivo* imaging, we found that the NF-κB activities in brain, liver, and intestine were significantly evoked by ionizing radiation. We therefore elucidated the gene expression profiles of brain, liver, and intestine by transcriptomic analysis. In a total 29,922 genes, the transcripts of 430, 1,169, and 1,309 genes in brain, liver, and intestine, respectively, passed the aforementioned criteria and were selected for further KEGG pathway classification. Table 1 shows that 26 pathways were significantly regulated in at least one organ after irradiation. The half of pathways was associated with metabolism, while others were related to immune system, cell growth and death, signal transduction, and genetic information process. Among 26 pathways, 5 metabolic pathways, including arginine and proline metabolism, citrate cycle, glycerolipid metabolism, oxidative phosphorylation and pyruvate metabolism, and 2 genetic inflammation processes, such as proteasome and ribosome pathways, were significantly altered in all these organs. In addition to the commonly regulated pathways, there were several pathways significantly and specifically regulated in one of these organs. These pathways included gap junction in the brain, glycerophospholipid metabolism and complement-coagulation cascades in the liver, and fatty acid metabolism, N-glycan biosynthesis, and hematopoietic cell lineage in the intestine. These findings indicated that ionizing radiation altered several pathways associated with metabolism, immune system, cell growth and death, signal transduction, and genetic information process. Moreover, pathways regulated by ionizing radiation displayed an organ-specific manner in intestine, brain, and liver.

**Table 1:** KEGG pathway analysis of genes in organs at 3 h after ionizing radiation.

KEGG pathway^a^	*p* value^b^ (total/up/down)^c^		
	Intestine	Brain	Liver
**Metabolism**			
Arginine and proline metabolism	0.01130 (52/0/2)	0.00338 (52/3/1)	0.02077 (52/1/2)
Bile acid biosynthesis	2.9×10^−6^ (34/3/2)	0.68843 (34/0/0)	0.01253 (34/4/3)
Butanoate metabolism	0.00250 (41/1/3)	0.14750 (41/1/0)	0.00391 (41/4/6)
Citrate cycle (TCA cycle)	0.00078 (24/1/3)	0.01938 (24/0/0)	0.00043 (24/1/1)
Fatty acid metabolism	0.00003 (36/4/3)	0.23500 (36/0/0)	0.14559 (36/3/1)
Glycerolipid metabolism	6.0×10^−6^ (38/2/4)	0.01790 (38/3/1)	0.03965 (38/3/6)
Glycerophospholipid metabolism	0.42737 (51/0/0)	0.13926 (51/0/0)	0.02609 (51/2/5)
Linoleic acid metabolism	0.00998 (40/1/2)	0.99948 (40/0/1)	0.00071 (40/4/3)
Lysine degradation	0.68298 (69/1/0)	0.00378 (69/10/1)	0.04571 (69/5/6)
Metabolism of xenobiotics by cytochrome p450	1.5×10^−7^ (52/2/6)	0.61449 (52/0/2)	0.00068 (52/3/6)
N-Glycan biosynthesis	0.00628 (32/0/1)	0.08642 (32/1/0)	0.25735 (32/0/1)
Oxidative phosphorylation	7.7×10^−13^ (101/2/13)	3.6×10^−16^ (101/4/15)	5.2×10^−8^ (101/3/4)
Pyruvate metabolism	0.00003 (31/3/0)	0.00632 (31/2/2)	0.00935 (31/3/3)
Tryptophan metabolism	0.02084 (59/4/2)	0.06264 (59/1/1)	0.03921 (59/3/5)
Valine, leucine and isoleucine degradation	0.01356 (41/3/1)	0.26859 (41/0/0)	0.00305 (41/2/5)
**Immune system**			
B cell receptor signaling pathway	0.01122 (64/3/1)	0.00713 (64/4/0)	0.43761 (64/0/0)
Complement and coagulation cascades	0.09729 (68/0/3)	0.57659 (68/0/1)	0.02019 (68/4/2)
Hematopoietic cell lineage	0.00553 (82/4/2)	0.15968 (82/2/0)	0.48065 (82/6/2)
**Cell growth and death**			
Apoptosis	0.01736 (80/6/1)	0.03479 (80/5/2)	0.73307 (80/0/0)
Gap junction	0.16539 (81/1/0)	0.00005 (81/9/2)	0.11232 (81/4/3)
Regulation of actin cytoskeleton	0.00419 (193/2/4)	7.3×10^−6^ (193/12/7)	0.08328 (193/9/9)
**Signal transduction**			
Insulin signaling pathway	0.05099 (77/1/0)	0.00004 (77/14/0)	0.01390 (77/2/10)
MAPK signaling pathway	0.01767 (248/5/5)	0.00006 (248/13/12)	0.33913 (248/3/6)
PPAR signaling pathway	0.00409 (70/5/1)	0.17488 (70/2/0)	0.00117 (70/5/5)
**Genetic information process**			
Proteasome	0.000547 (30/0/3)	0.01046 (30/1/2)	0.00001 (30/4/2)
Ribosome	4.7×10^−18^ (80/2/17)	6.7×10^−17^ (80/0/24)	2.4×10^−6^ (80/13/2)

a Genes with fold changes >1.8 or <−1.8 were analyzed by KEGG pathways.

b *p* values were calculated by the geneSetTest function implemented in the limma package.

c Total number of genes in this pathway / Number of upregulated genes in this pathway/Number of downregulated genes in this pathway.

**Figure 4:**
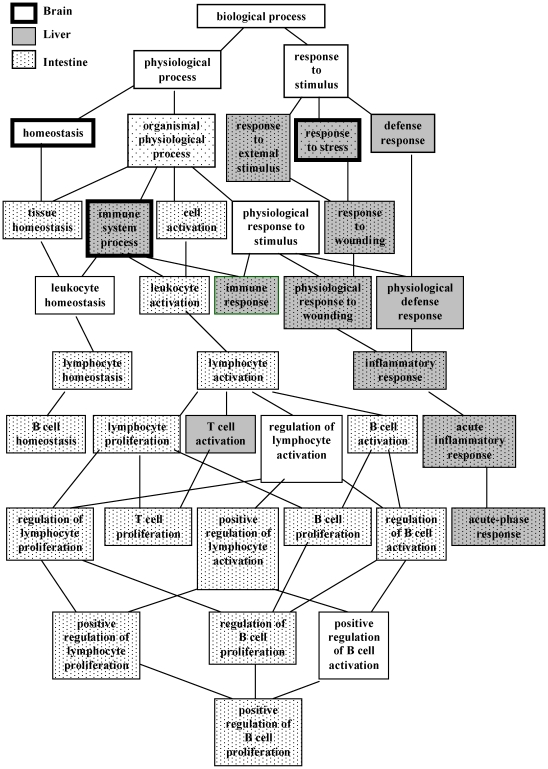
GO analysis of organs after irradiation. Differential expressed genes responsive to ionizing radiation were organized using Gene Ontology Tree Machine. The significantly regulated GO categories in brain, liver, and intestine are indicated.

We further used the WebGestalt tool on Gene Ontology Tree Machine web site to annotate these genes and to get an overview of cellular physiological status altered by ionizing radiation in these organs. GO categories were considered if they contained at least 2 genes and their *p*-values were below 0.01. As shown in Figure 4, ionizing radiation affected different GO terms in different organs. There were several GO categories specifically altered in the intestine. Two GO terms, including T cell activation and homeostasis, were specifically regulated in the liver and brain, respectively. However, two GO terms, including immune system process and response to stress, were altered commonly in these organs. The expression levels of differentially expressed genes belonging to the GO categories of “immune system process” and “response to stress” are shown in Table 2. The half of genes was involved in antigen processing and presentation, chemokine or B cell receptor signaling pathway, and complement-coagulation cascades. Additionally, some genes were associated with cell growth and death, transcription factors, and metabolism.

**Table 2:** The expression levels of genes belonging to the GO categories of “immune system process” and “response to stress”.

Gene description	Fold changes^a^		
	Intestine	Brain	Liver
**Antigen processing and presentation**			
Heat shock protein 1 (chaperonin)	−5.63±1.20	1.22±0.03	−1.43±0.35
Heat shock protein 8	−1.17±0.91	−1.20±0.75	−2.80±1.50
**Chemokine or B cell receptor signaling pathway**			
CD79B antigen	−5.80±1.13	1.15±0.07	1.15±0.07
CD 81 antigen	3.00±0.35	1.40±0.28	1.50±0.57
Chemokine (CC motif) ligand 5	−2.50±0.70	−0.55±0.64	−1.15±0.07
Chemokine (CC motif) ligand 19	−1.90±0.14	1.10±0.03	−1.30±0.28
Chemokine (CC motif) ligand 20	−2.85±0.21	0.37±1.18	1.13±0.06
Chemokine (CXC motif) ligand 1	1.85±0.49	1.15±0.07	7.05±2.62
Chemokine (CXC motif) ligand 9	1.85±0.49	−1.23±0.23	−1.53±0.45
Chemokine (CXC motif) ligand 12	−2.17±0.21	1.15±0.21	−1.10±0.10
Chemokine (CXC motif) ligand 16	1.30±0.82	1.40±0.85	1.93±0.78
Conserved helix-loop-helix ubiquitous kinase	−2.17±0.06	1.10±0.14	−1.25±0.21
Cysteine-rich protein 3	1.65±0.78	−0.80±0.35	−1.55±0.64
Interleukin 22	−0.60±2.26	−3.50±0.57	1.30±0.20
Jagged 1	2.40±0.28	1.07±0.06	1.13±0.15
Kinase insert domain protein receptor	2.25±0.07	1.13±0.15	−1.33±0.25
S100 calcium binding protein A9 (calgranulin B)	1.83±0.29	1.30±0.04	1.80±0.60
Signal transducer and activator of transcription 3	2.40±0.53	1.42±0.49	−1.43±0.35
Tumor necrosis factor receptor superfamily, member 13c	−2.03±0.06	1.03±0.06	1.13±0.06
**Complement and coagulation cascades**			
C-reactive protein, pentraxin-related	2.30±0.87	−2.50±1.85	1.40±0.20
Complement component 1, q subcomponent, alpha polypeptide	1.87±1.21	−1.60±0.50	−1.20±0.10
Fibrinogen, gamma polypeptide	−3.13±1.10	−1.05±0.07	2.60±1.27
Hemolytic complement	−2.20±1.13	1.13±0.06	−1.70±0.28
Serine (or cysteine) peptidase inhibitor, clade A, member 1a	−2.73±0.81	−1.23±0.15	−1.17±0.15
Serine (or cysteine) peptidase inhibitor, clade C (antithrombin), member 1	−3.70±1.57	1.10±0.14	−1.73±0.45
Serum amyloid A 2	−2.20±0.60	1.20±0.28	13.37±5.35
Serum amyloid A 3	2.47±0.64	1.30±0.28	11.65±5.02
**Cell growth and death**			
Alpha 1 microglobulin/bikunin	−3.13±1.01	−1.30±0.28	−1.28±0.03
B-cell translocation gene 2, anti-proliferative	2.77±0.74	1.25±0.21	1.27±0.25
Bcl2-associated X protein	3.87±1.40	1.25±0.21	1.53±0.15
Caspase 8	−2.73±0.76	−1.03±0.04	−1.35±0.35
Cyclin-dependent kinase inhibitor 1A (P21)	2.80±0.69	1.60±0.40	1.40±0.42
Gap junction membrane channel protein alpha 1	−0.70±2.97	−2.75±0.35	1.08±0.04
Growth arrest and DNA-damage-inducible 45 alpha	−6.10±3.42	1.11±0.01	−2.08±0.04
Guanylate nucleotide binding protein 1	2.20±0.60	1.30±0.20	−1.65±0.78
Mitogen activated protein kinase 1	−2.80±0.72	0.40±2.10	−1.15±0.21
Mitogen activated protein kinase 3	−1.87±0.76	−1.23±0.15	−2.20±0.10
Nucleophosmin 1	−2.90±1.35	−1.30±0.28	1.60±0.28
Trefoil factor 1	−2.27±0.61	−1.20±0.10	−1.55±0.78
**Transcription factors**			
Early growth response 1	−1.23±1.00	−1.10±0.53	−2.20±1.27
Interferon regulatory factor 7	1.40±1.20	−3.30±1.00	1.08±0.04
**Metabolism**			
Adenosine deaminase	−2.93±0.76	1.20±0.28	1.05±0.07
Apolipoprotein E	−1.80±0.53	−1.77±0.45	−1.30±0.28
Fatty acid binding protein 4, adipocyte	1.23±0.80	1.00±0.03	3.37±2.35
Ferritin heavy chain 1	−2.67±1.10	−2.17±0.85	1.67±0.25
Glutathione peroxidase 1	−3.07±0.83	−2.10±0.40	−1.40±0.14

a Values are mean ± standard error (*n* = 6).

### Verification of the expression levels of ionizing radiation-regulated genes by qPCR

Transcriptomic analysis showed that ionizing radiation altered the pathways associated with metabolism and immune system. We therefore validated the expression levels of immuno-related and metabolism-related genes by qPCR analysis. As shown in Table 3, the expression levels of Ccl5 and Ccl20 genes in the intestine were down-regulated, and the expression levels of Jagged1 gene in the intestine, and SAA2 and SAA3 genes in the liver were upregulated by ionizing radiation, which were in agreement with the microarray data.

**Table 3:** Expression levels of Ccl5, Ccl20, Jagged1, SAA2, and SAA3 genes by qPCR.

Sample	Average C_T_ of target	Average C_T_ of GAPDH	ΔC_T_ a	ΔΔC_T_ b	Relative to Mock
**Intestine**					
Ccl5					
Mock	26.88±0.11	20.03±0.10	6.85±0.15	0.00±0.15	1.00
Ionizing radiation	26.19±0.04	19.02±0.02	7.17±0.05	0.31±0.05	0.80
Ccl20					
Mock	22.36±0.04	19.73±0.01	2.62±0.05	0.00±0.05	1.00
Ionizing radiation	22.92±0.03	19.92±0.03	2.99±0.05	0.36±0.05	0.77
Jagged1					
Mock	27.13±0.02	19.73±0.01	7.40±0.03	0.00±0.03	1.00
Ionizing radiation	26.80±0.10	19.92±0.03	6.88±0.10	−0.52±0.10	1.43
**Liver**					
SAA2					
Mock	27.81±0.10	18.41±0.03	9.40±0.11	0.00±0.11	1.00
Ionizing radiation	24.49±0.10	18.64±0.04	5.85±0.11	−3.55±0.11	11.71
SAA3					
Mock	27.66±0.11	19.73±0.01	7.93±0.11	0.00±0.11	1.00
Ionizing radiation	26.82±0.08	19.92±0.03	6.90±0.09	−1.03±0.09	2.04

a The ΔC_T_ value is determined by subtracting the average GAPDH C_T_ value from the average target gene C_T_ value. The standard deviation of the difference is calculated from the standard deviations of the target gene and GAPDH.

b The calculation of ΔΔC_T_ involves subtraction by the ΔC_T_ calibrator value. This is a subtraction of an arbitrary constant, so the standard deviation of ΔΔC_T_ is the same as the standard deviation of the ΔC_T_ value.

## Discussion

NF-κB can function as a sensor to detect cell responses to various stimuli, such as ionizing radiation. Therefore, NF-κB-dependent luminescent signal was used as a guide to indicate which organs were affected by ionizing radiation in this study. Irradiation significantly increased NF-κB-dependent luminescent signals in brain, liver, and intestine, suggesting that irradiation evoked significant biological events in these organs. Microarray tool was further performed to elucidate the biological events evoked by ionizing radiation. Network analysis showed that some of the irradiation-altered genes were directly linked to NF-κB (Figure S2). Others were directly linked to other transcription factors like signal transducer and activator of transcription 3, and these transcription factors were further linked to NF-κB. Therefore, these data indicated that the expression levels of genes were directly or indirectly regulated by NF-κB activity. Moreover, these findings also suggested that NF-κB can be a sensor to sense various biological events in the animals after exposure.

In this study, we applied NF-κB bioluminescent imaging to monitor the *in vivo* NF-κB activity in mice after irradiation. A single dose of 8.5 Gy was chosen because it is the lethal dose for adult mice and it is often applied for total-body irradiation [23], [24]. Irradiation induced a maximal activation of NF-κB at 3 h. Irradiation increased the NF-κB-dependent bioluminescence in most organs because it is well known that NF-κB activity can be induced by various stimuli, including irradiation [5]. Despite this, irradiation increased different levels of luminescent signals in different organs. For examples, highly NF-κB-dependent luminescent signals were observed in brain, liver, and intestine, while moderate signals were observed in heart, lung, spleen, kidney, and testis. These findings suggested that ionizing radiation induced an organ-specific activation of NF-κB *in vivo*, which was in agreement with previous observations [13], [25]. However, there were discrepancies between present findings and previous reports. Previous reports indicated that irradiation induces NF-κB activities in spleen, lymph node, bone marrow, and intestine, but not in liver, lung, colon, and brain. Our data showed that ionizing radiation induced highly NF-κB-driven bioluminescence signals in brain, liver, and intestine, and moderate signals in heart, lung, spleen, kidney, and testis. The sensitivity of assay (NF-κB DNA-binding ability in previous reports and NF-κB-driven luciferase activity in this report), mice strains (C57BL/6J in previous reports and FVB strain in this report), and dose of irradiation might contribute to the differences in the results of experiments. For examples, when mice are exposed to a clinically relevant dose (≤2 Gy) of total body irradiation, a significant activation of NF-κB is found in spleen, lymph node, bone marrow, and small intestine [13]. However, when mice are exposed to a super lethal dose (20 Gy) of total body irradiation, a significant activation of NF-κB is found in liver and kidney [26]. The tissue-specific manner of irradiation might result from the sensitivity to irradiation toxicity in different organs. Microenvironment or inherent tissue-specific intracellular signaling pathways might also contribute to the tissue-specificity of irradiation. In comparison with previous studies, we newly identified that a significant activation of NF-κB was observed in brain after exposure to 8.5 Gy total body irradiation and the NF-κB activation was observed in oligodendrocytes. Recent study indicates that irradiation induces regionally specific alterations in pro-inflammatory environments in rat brain and this has been implicated in the onset and progression of neurological disorders [27]. Moreover, low-dose irradiation causes minimal histopathologic change; however, it can elicit variable degrees of cognitive dysfunction that are associated with the depletion of neural stem cells [28]. Since NF-κB functions as a sensor that can detect ionizing radiation-induced tissue damage [13], NF-κB activation found in the brain might reasonably explain the irradiation-induced brain injury.

NF-κB bioluminescent imaging showed that NF-κB activities in brain, liver, and intestine were significantly induced by irradiation. The gene expression profiles of these organs were further analyzed by DNA microarray. DNA microarray is a popular research and screening tool for differentially expressed genes [29]. Our data showed that ionizing radiation altered gene expression signatures in a tissue-specific manner. Markedly different gene expression responses between kidney and brain have been reported previously [9]. Gap junction and ribosome were regulated in the brain after irradiation with 8.5 Gy at 3 h (this study) or 20 Gy at 24 h [10]. Lipid metabolism and cell progression pathways were influenced in the liver after irradiation with 8.5 Gy at 3 h (this study) or 1 Gy at 3 h [11]. Signal transductions, including epidermal growth factor and insulin-like growth factor, are altered in liver cells exposed to α-particles [12], while we newly identified that insulin and peroxisome proliferator-activated receptor (PPAR) signal transduction pathways were significantly regulated in the liver. Radiation-induced small intestine injury is characterized by cell loss in the progenitor cell compartment and a dose-dependent loss of barrier properties [30]. Loss of the intestinal absorptive surface and a consequent decrease in active transport leads to the change in intestinal absorption after irradiation. Previous studies indicated that abdominal irradiation influences the uptake of carbohydrates, amino acids, fatty acids, cholesterols, and bile acids into the jejunum [31]–[33]. By KEGG pathway analysis, we found for the first time that nutrient metabolisms, including lipids, carbohydrates, amino acids and energy, and metabolism-related pathway, such as PPAR signaling pathway, were significantly altered in the intestine after irradiation. These findings suggested that, in addition to the absorption, radiation might influence the nutrient metabolism in the intestine. Overguard and Mutsui [33] have shown that the absorption insufficient in the small intestine is only present acutely after irradiation and no late abnormalities are seen 12 months later. Whether the irradiation led to the long-term influence on the nutrient metabolism in the intestine remained to be clarified.

The expression levels of some immunomodulatory genes, including Ccl5, Ccl19, Ccl20 and cluster of differentiation 79B (CD79B) antigen, in the intestine were downregulated after irradiation. Chemokine (CC motif) ligands are potent chemoattractants for monocytes, memory helper T-lymphocytes, eosinophils, basophils, mast cells, and dendritic cells [34]. CD79B antigen is a B-lineage-specific member of the immunoglobulin superfamily. It is consisted of a single extracellular immunoglobulin-like domain and an intracytoplasmic tail that contains a motif involved in lymphocyte activation [35]. Downregulation of chemokine (CC motif) ligands and CD79B antigen gene expressions suggested that irradiation might suppress the inflammation or immune response of intestine. The expression level of Jagged1 gene was upregulated in the intestine after irradiation. Jagged1 is a ligand for canonical Notch signaling [36]. Notch signaling is required for the control of intestinal epithelial cells self-renewal and the allocation of these cells to specific differentiation lineages [37]. The expression of Jagged1 is restricted to enteroendocrine cells and undetectable in the mucosa of the normal human small and large intestine [38], [39]. Elevated Jagged1 expression in the intestine after irradiation might activate Notch signaling and, in turn, amplify the intestinal progenitor pool and inhibit cell proliferation. Additionally, recent study indicates that Notch activation, accomplished by Wnt signaling-mediated upregulation of Jagged1, is required for tumorigenesis in the intestine [39], [40]. Therefore, irradiation altered the expression levels of Ccl5, Ccl19, Ccl20, CD79B antigen, and Jagged 1 genes, suggesting that these genes might be involved in the radiation enteropathy.

The expression levels of immunomodulatory genes, including chemokine (CXC motif) ligand 1 (Cxcl1) and S100 calcium binding protein A9 (calgranulin B) (S100A9), in the liver were upregulated. Cxcl1 is a small cytokine belonging to the CXC chemokine family. Cxcl1 is expressed in macrophages, neutrophils, and epithelial cells, and has neutrophil chemoattractant activity [41]. S100A9 is a small calcium-binding protein that is highly expressed in neutrophils and monocytes. It is at a high level in the extracellular milieu during inflammatory conditions and involved in neutrophil migration to inflammatory sites [42]. Fatty acid binding protein 4 (FABP4), SAA2, and SAA3 were also upregulated by irradiation in the liver. FABP4 has an important role in regulating systemic insulin resistance and lipid metabolism [43]. Moreover, the metabolic-inflammatory pathway cross-regulation by FABPs contributes to the adaptive immune responses and the subsequent autoimmune inflammation [44]. SAA proteins are a family of apolipoproteins associated with the high-density lipoprotein in plasma. SAA2 and SAA3 are regulated in the liver by the proinflammatory cytokines, such as interleukin-1, interleukin-6, and tumor necrosis factor-α [45]. SAA2 are induced locally and systematically in mice under acute inflammatory conditions. Additionally, SAAs are also involved in lipid metabolism [46]. FABP4, SAA2, and SAA3 are participated in both inflammation and lipid metabolism, suggesting that irradiation might affect the cross pathways of metabolism and inflammation in the liver.

In conclusion, we applied NF-κB bioluminescent imaging-guided transcriptomic analysis to evaluate the host responses to irradiation. Irradiation induced an acute activation of NF-κB at 3 h. Microarray analysis of brain, liver, and intestine showed that irradiation altered several pathways associated with metabolism and immune system. GO analysis further showed that irradiation altered two common GO terms, including immune system process and response to stress, in these organs. This report described the comprehensive evaluation of host responses to irradiation exposure. Our findings provided the fundamental impacts into the radiation-affected NF-κB activity and transcriptomic pattern in the whole body. Moreover, novel targets involved in radiation injury were also suggested.

## Supporting Information

Figure S1 NF-κB-dependent bioluminescence in living mice.
(PDF)

Figure S2 Network analysis of irradiation-affected genes.
(PDF)
