# Supplementary material for: Comprehensive Assessment of Host Responses to Ionizing Radiation by Nuclear Factor-κB Bioluminescence Imaging-Guided Transcriptomic Analysis
Source: PLoS One. 2011 Aug 24;6(8):e23682. doi: 10.1371/journal.pone.0023682 (PMC3161058; doi:10.1371/journal.pone.0023682)
Supplement: Figure S1 — NF-κB-dependent bioluminescence in living mice. (PDF) [file pone.0023682.s001.pdf]

Figure S1. NF-əB-dependent bioluminescence in living mice. Transgenic mice were exposed to ionizing radiation and imaged in dorsal (A) and ventral positions (B) at 0 h, 1 h, and 3 h. The color overlay on the image represents the photons/sec emitted from the animal, as indicated by color scale. Photos from three independent mice each group were shown.

(A)

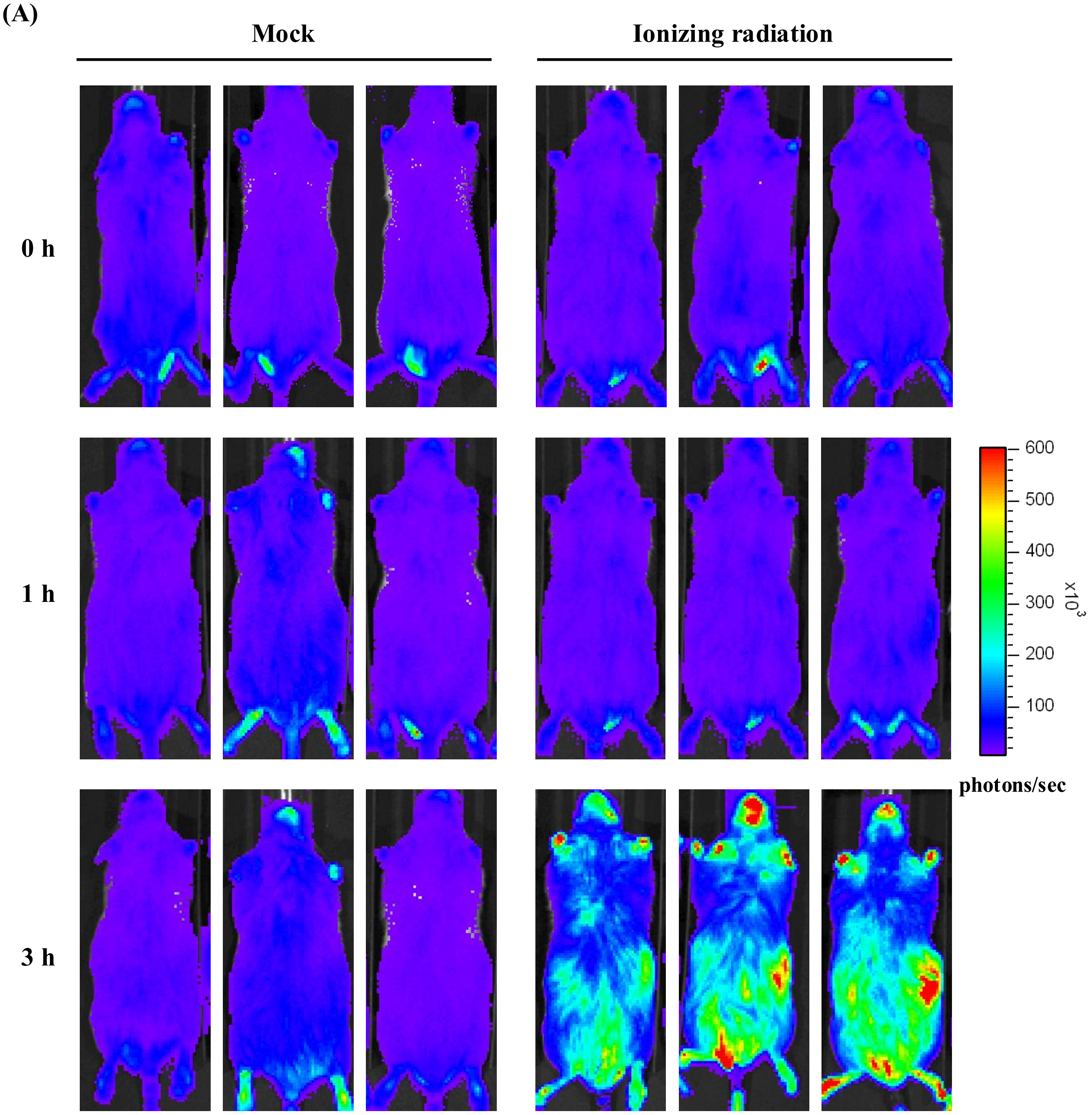

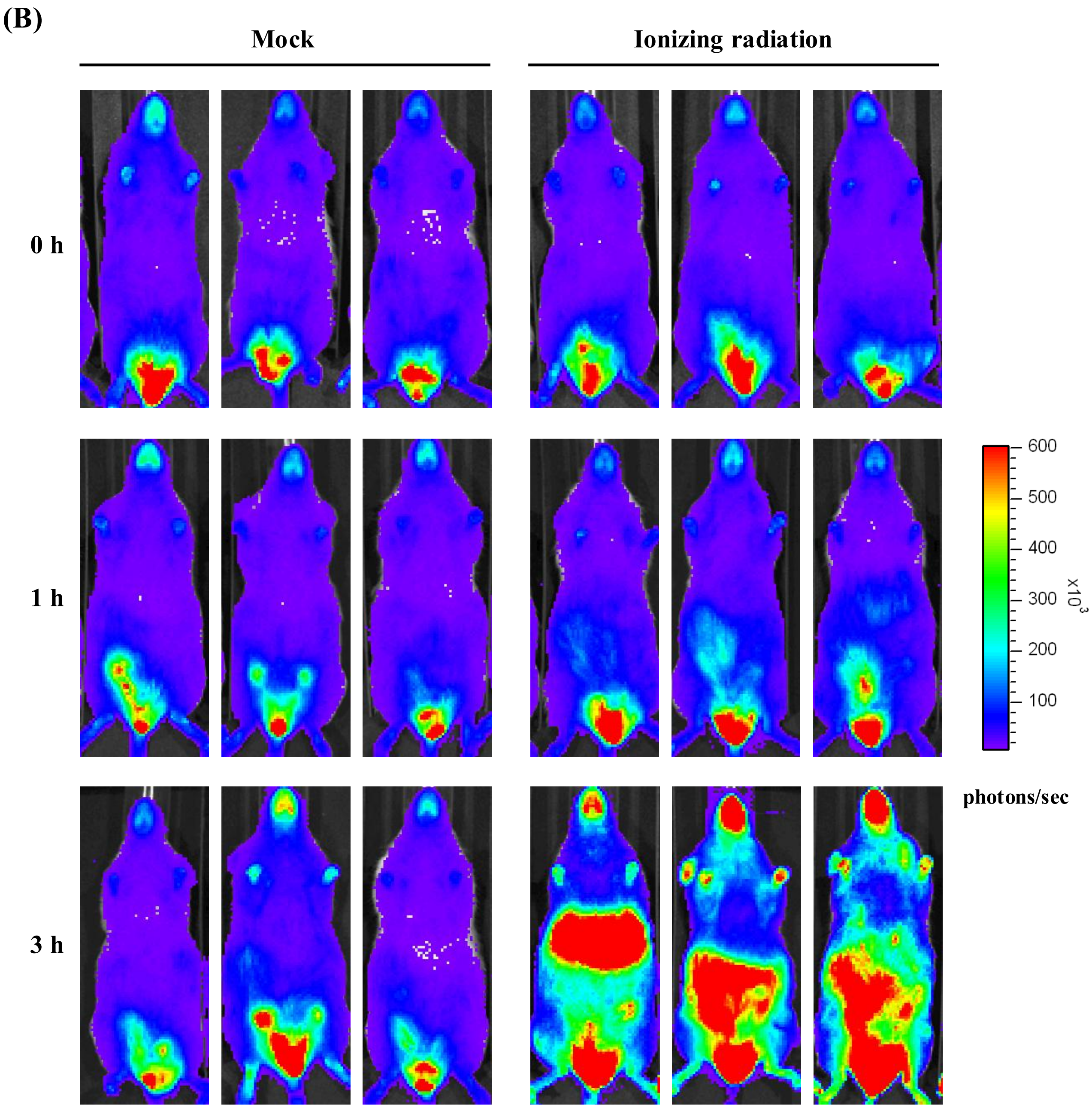
(B)
Mock
Ionizing radiation
0 h
1 h
3 h
600
500
400
300
200
100
x10³
photons/sec
