# Supplementary material for: Comprehensive Assessment of Host Responses to Ionizing Radiation by Nuclear Factor-κB Bioluminescence Imaging-Guided Transcriptomic Analysis
Source: PLoS One. 2011 Aug 24;6(8):e23682. doi: 10.1371/journal.pone.0023682 (PMC3161058; doi:10.1371/journal.pone.0023682)

Figure S2. Network analysis of irradiation-affected genes. Differentially expressed genes belonging to the Go categories of "immune system process" and "response to stress" were used as input genes for the generation of biological network using Transcription Regulation algorithm in MetaCore^{TM} Analytical Suite (GeneGo Inc., St. Joseph, MI, USA). Green links between genes represent positive effects between the genes. Red links between genes represent negative effects between the genes. The grey arrows represent technical links between the genes. Key to symbols: represents a generic enzyme; represent a protein kinase; represents a generic protease; represents a receptor ligand; represents a receptor with enzyme activity; represents a generic binding protein; represents a transfactor.

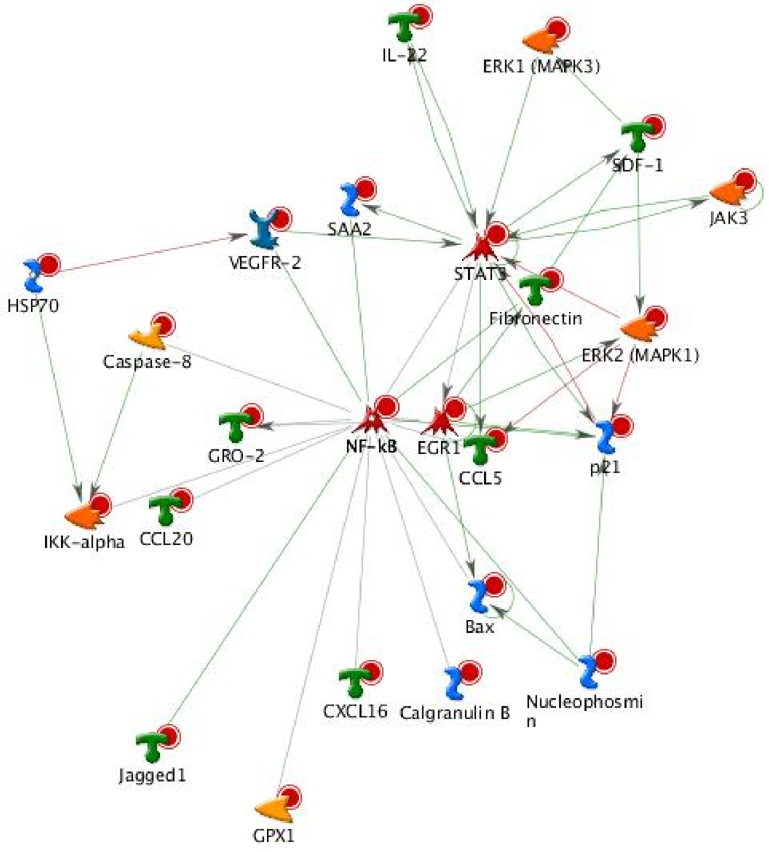

Supplement: Figure S2 — Network analysis of irradiation-affected genes. (PDF) [file pone.0023682.s002.pdf]
